# Age and Mutations as Predictors of the Response to Immunotherapy in Head and Neck Squamous Cell Cancer

**DOI:** 10.3389/fcell.2020.608969

**Published:** 2020-12-09

**Authors:** Yueming Zhang, Anqi Lin, Yonghe Li, Weimin Ding, Hui Meng, Peng Luo, Jian Zhang

**Affiliations:** ^1^Department of Oncology, Zhujiang Hospital, Southern Medical University, Guangzhou, China; ^2^Department of Otolaryngology-Head and Neck Surgery, Zhujiang Hospital, Southern Medical University, Guangzhou, China

**Keywords:** head and neck cancer, immunotherapy, predictor, age, gene mutation, immune microenvironment

## Abstract

The immunosuppressive tumor microenvironment plays an essential role in the treatment of head and neck squamous cell carcinoma (HNSC). Compared to traditional chemoradiotherapy, immune checkpoint inhibitors (ICIs) have become increasingly important in HNSC therapy. Prior studies linked the efficacy of ICIs to PD-L1, microsatellite instability (MSI), HPV infection, tumor mutation burden (TMB), and tumor lymphocyte infiltration in patients with HNSC, but further verification is needed. Additional predictors are needed to recognize HNSC patients with a good response to ICIs. We collected the clinical information and mutation data of HNSC patients from Memorial Sloan Kettering Cancer Center (MSKCC) and The Cancer Genome Atlas (TCGA) databases to generate two clinical cohorts. The MSKCC cohort was used to recognize predictors related to the efficacy of ICIs, and the TCGA cohort was used to further examine the immune microenvironment features and signaling pathways that are significantly enriched in the subgroups of predictors. Multivariate Cox regression analysis indicated that age (HR = 0.50, *p* = 0.014) and ARID1A (HR = 0.13, *p* = 0.048), PIK3CA (HR = 0.45, *p* = 0.021), and TP53 (HR = 1.82, *p* = 0.035) mutations were potential predictors for ICI efficacy in HNSC patients. Age > 65 years and ARID1A or PIK3CA mutations correlated with good overall survival (OS). TP53 mutant-type (MT) patients experienced a worse prognosis than TP53 wild-type (WT) patients. The subgroups associated with a good prognosis (age > 65 years, ARID1A-MT, and PIK3CA-MT) universally had a high TMB and increased expression of immune checkpoint molecules. Although TP53-MT was associated with a high TMB, the expression of most immune checkpoint molecules and immune-related genes was lower in TP53-MT patients than TP53-WT patients, which may reflect low immunogenicity. Pathways related to the immunosuppressive tumor microenvironment were mostly enriched in the subgroups associated with a poor prognosis (age ≤ 65 years, low TMB, ARID1A-WT, PIK3CA-WT, and TP53-MT). In conclusion, the factors age > 65 years, PIK3CA-MT, and ARID1A-MT predicted favorable efficacy for ICI treatment in HNSC patients, and TP53 mutation was a negative predictor.

## Introduction

Head and neck cancers include tumors arising in the lip, oral cavity, pharynx, larynx, and paranasal sinuses, and occult primary cancer, salivary gland cancer, and mucosal melanoma (National Comprehensive Cancer Network [NCCN], 2019). Squamous cell carcinoma is the main histological type, and it accounts for more than 90% of these tumors (National Comprehensive Cancer Network [NCCN], 2019). Head and neck squamous cell carcinoma (HNSC) is a common malignant tumor. According to the GLOBOCAN 2018 estimates of cancer incidence and mortality, there were approximately 830,000 new cases of tumors arising in the lip, oral cavity, pharynx and larynx, which accounted for 4.6% of the new global cancer cases. Approximately 450,000 patients died of these tumors, which accounted for 4.6% of global cancer-related deaths (Bray et al., 2018). Patients who present with early stage disease (stage I or II) may be treated with surgery or radiotherapy alone or concurrent chemoradiotherapy (National Comprehensive Cancer Network [NCCN], 2019). The preferred scheme for patients with locally advanced disease is concurrent chemoradiotherapy (National Comprehensive Cancer Network [NCCN], 2019). However, the therapeutic efficacy of systemic therapy is limited. The 5 years overall survival (OS) rate of HNSC patients receiving concurrent chemoradiotherapy is approximately 50% (Lin et al., 2016), and the median OS time is 66.3 months (Stokes et al., 2017).

The immunosuppressive tumor microenvironment is an important feature of HNSC (Oddone et al., 2009). Previous studies indicated that HPV-positive HNSC displayed the highest levels of immune cell infiltration compared to other cancer types (Mandal et al., 2016). Notably, these patients also presented high levels of immunosuppression (Mandal et al., 2016). Tumor cells evade immunosurveillance and inhibit the activation and function of immune cells by inducing the production of immunosuppressive factors, reducing tumor immunogenicity, and recruiting immune cells with immunosuppressive functions (De Costa and Young, 2011; Moy et al., 2017; Concha-Benavente et al., 2018; Peltanova et al., 2019; Plzak et al., 2019). Immune checkpoint inhibitors (ICIs) recently, expanded our horizons of cancer therapy. ICIs targeting CTLA-4 and PD-(L)1 led to dramatic advances in non-small cell lung cancer (Reck et al., 2016; Sui et al., 2018). Immune checkpoint blockade enables immune cells to regain the ability to recognize tumor cells (Seidel et al., 2018). The application of nivolumab and pembrolizumab targeting PD-1 in HNSC patients was approved by the FDA (National Comprehensive Cancer Network [NCCN], 2019). Burtness et al. (2019) performed a phase III clinical trial of HNSC patients with recurrent or metastatic diseases and showed that pembrolizumab monotherapy or pembrolizumab combined with chemotherapy effectively prolonged the OS of HNSC patients.

Although ICIs show great application prospects in the treatment of cancers (Reck et al., 2016; Bertucci and Goncalves, 2017; Kang et al., 2017; Sui et al., 2018; Fan et al., 2019), their efficacy is heterogeneous in different tumors and different patients with the same tumor. The identification of biomarkers of immunotherapy efficacy is urgently needed to further recognize individuals who are likely sensitive to immunotherapy. A large number of studies indicated that the expression of PD-L1 (Patel and Kurzrock, 2015; Chang et al., 2018), tumor mutation load (TMB) (Chan et al., 2019; Liu et al., 2019), microsatellite instability (MSI) (Chang et al., 2018; Luchini et al., 2019; Zhao et al., 2019), and mutations in specific genes (Dong et al., 2017a; Xiao et al., 2018) can predict the efficacy of ICIs. Some researchers also believe that the clinical characteristics of patients, such as sex (Conforti et al., 2018; Wu et al., 2018; Lin W. et al., 2020), and age (Kugel et al., 2018; Lin W. et al., 2020), are related to the efficacy of immunotherapy.

An increasing number of studies showed that driver gene mutations may cause differences in the efficacy of ICIs by affecting the tumor immune microenvironment (Huang et al., 2020; Lin A. et al., 2020; Lin W. et al., 2020; Niu et al., 2020; Yi et al., 2020; Zhang et al., 2020). Dong et al. (2017a) indicated that non-small cell lung cancer patients with EGFR mutations responded poorly to PD-(L)1 inhibitors, which may be related to reduced CD8^+^ T lymphocyte infiltration and low immunogenicity. Dong et al. (2017b) also showed that lung adenocarcinoma patients with co-mutation of TP53 and KRAS had a high TMB, high PD-L1 expression and high levels of CD8^+^ T lymphocyte infiltration, which may promote the response to ICI therapy by affecting the cell cycle, DNA replication, and DNA repair. Wang et al. (2019) and Wu et al. (2019) suggested that patients with TET1 or POLE/POLD1 gene mutations were likely to benefit from ICI therapy.

However, heterogeneity exists in different tumor types, and these biomarkers may not reflect the sensitivity of all patients to ICI treatment. Under the background of HNSC, related studies noted that some characteristics, such as PD-L1 (Ferris et al., 2016), MSI (Tardy et al., 2018), HPV infection (Zandberg et al., 2019), TMB (Hanna et al., 2018), and tumor lymphocyte infiltration (Hanna et al., 2018), may be predictors of immunotherapy efficacy. The different outcomes of HNSC patients with or without HPV infection may be attributed to the distinct immune cell infiltration (Cillo et al., 2020). More large-scale studies are needed to verify their reliability and stability in clinical applications. We need to screen additional biomarkers to promote the clinical application of immunotherapy. There may be a relationship between different biomarkers, and the combination of biomarkers may provide new insights into patient sensitivity toward ICIs (Luchini et al., 2019; Zhao et al., 2019).

The present study detected markers of immunotherapy efficacy in HNSC patients using their clinical characteristics and gene mutation data. We examined the relationship between the tumor immune microenvironment and relevant clinical characteristics or gene mutations in immune gene activation, immune-related cell infiltration, markers of immune cell exhaustion and the activation of pathological pathways. Our results provide insight into how clinical characteristics and gene mutations affect ICI efficacy from the perspective of the immune microenvironment to provide new ideas for the study of the immunotherapy mechanisms related to HNSC and guide treatment selection or improve responses to ICIs.

## Materials and Methods

### Clinical Cohorts

We analyzed an immunotherapeutic cohort from Memorial Sloan Kettering Cancer Center (MSKCC) described by Samstein et al. (2019), of which 138 HNSC patients were treated with ICIs (Supplementary Figure S1). Samples with somatic mutation and clinical data (*n* = 129) were selected to evaluate the relationship between age, gene mutations, and the prognosis of HNSC patients treated with ICIs. A detailed flow chart of the analysis is shown in Supplementary Figure S1. The somatic mutation data in the cohort were derived from HNSC patients receiving anti-PD-(L)1 monotherapy or anti-PD-(L)1 combined with anti-CTLA-4 therapy whose DNA was sequenced using the Memorial Sloan Kettering-Integrated Mutation Profiling of Actionable Cancer Targets (MSK-IMPACT) panel.

The R package TCGAbiolinks (Colaprico et al., 2016) was used to download the most recent clinical data and sample information (mRNA expression profile, somatic mutation data) of HNSC patients in The Cancer Genome Atlas (TCGA) database from the Genomic Data Commons^1^. Gene expression in the TCGA-HNSC dataset was in units of pan-cancer normalized log2 [fragments per kilobase of exon model per million mapped fragments (FPKM) + 1]. The processes of tumor RNA extraction, mRNA library preparation, sequencing, quality control, and subsequent data processing for quantitative gene expression in TCGA-HNSC samples were described in the literature (Cancer, 2014).

### Identification of Survival-Related Age Group and Establishment of Prognostic Gene Mutations

The clinical data and mutation information of the MSKCC cohort were used to identify the age group and gene mutations associated with the prognosis of HNSC patients. The optimal thresholds of age and tumor mutation count grouping were based on the surv_cutpoint function of the R package survival. Age, tumor mutation count, other clinical information, and gene mutations with a mutation frequency > 5 were included in univariate Cox regression analysis, in which factors with statistical significance (*p* < 0.1) and clinical factors were selected for the subsequent multivariate Cox regression analysis. Kaplan-Meier (KM) analysis and log-rank test were used to evaluate the prognosis of HNSC patients based on the variables identified as statistically significant (*p* < 0.1) in the univariate Cox regression analysis and clinical factors.

### Immune Characteristics and Tumor Immunogenicity Analysis

Immune-related genes and neoantigen load data of the TCGA-HNSC dataset were derived from Thorsson et al. (2018), and the expression levels of these genes were quantified as log2 (FPKM+1). TMB refers to the total number of substitution and insertion/deletion mutations per megabase in the exon coding region of the tumor genome (Yarchoan et al., 2017). Non-synonymous mutations were divided by 38 to quantify TMB in the TCGA and MSKCC cohorts (Chalmers et al., 2017).

EdgeR is a bioconductor package that provides methods to analyze the differential gene expression of RNA-seq data (Robinson et al., 2010). Our study used edgeR to calculate the log (fold change) and *p*-value of the differential expression of immune-related genes (Robinson et al., 2010), and these genes were visualized according to their functional classification.

### Functional and Pathway Enrichment Analyses

Gene set enrichment analysis (GSEA) identifies significantly enriched pathways by evaluating the differential gene expression in annotated gene set across subgroups of patients (Subramanian et al., 2005). The R package edgeR was used to standardize the gene expression data (raw count) of HNSC downloaded by TCGAbiolinks (Robinson et al., 2010). GSEA was performed using the R package clusterProfiler (Yu et al., 2012), and *p* < 0.05 was considered statistically significant in the Gene Ontology (GO), Kyoto Encyclopedia of Genes and Genomes (KEGG) and Reactome analyses. The GO, KEGG, and Reactome gene sets used for GSEA were derived from the Molecular Signatures Database (MSigDB) of the Broad Institute (Subramanian et al., 2005).

### Statistical Analysis

For groups with significant differences in the univariate Cox regression model, the Mann-Whitney U test was used to compare differences in TMB, neoantigen load and the mRNA expression of immune-related genes in the TCGA cohort. The KM analysis, log-rank test and univariate Cox proportional hazards regression analysis were used to assess OS in the MSKCC cohort. *P* < 0.05 was considered statistically significant, and all statistical tests were two-sided. R software (version 3.6.1) was used for statistical analyses. The R package ComplexHeatmap (Gu et al., 2016) was used to visualize the mutation and immune cell landscape. The R package ggplot2 (Wickham, 2009) was used to visualize bubble plots, violin plots, forest plots, alluvial plots, and volcano plots, and the R package trackViewer (Ou and Zhu, 2019) was used to visualize lollipop plots.

## Results

### Cox Regression Analysis

As shown in Supplementary Figure S1, our analysis consisted of two clinical cohorts. The MSKCC cohort (*n* = 129) was used to examine the relationship between clinical characteristics, gene mutations and the prognosis of HNSC patients. The clinical data and sample information of HNSC patients from the TCGA database were downloaded to establish the TCGA cohort (*n* = 489), which was used for the subsequent tumor immunogenicity analysis and GSEA.

**FIGURE 1:**
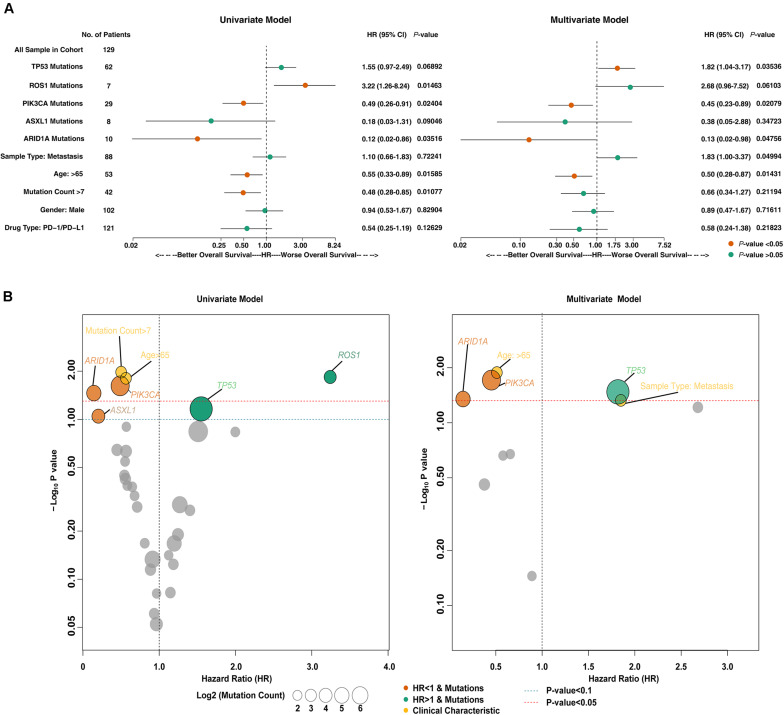
The predictive value of clinical characteristics and mutant genes on ICIs efficacy. **(A)** Forest plots for the results of the univariate (left panel) and multivariate (right panel) Cox regression analyses. In the univariate Cox regression analysis, the factors with a *p*-value less than 0.1 were age, mutation count, and mutations in ROS1, PIK3CA, ARID1A, TP53, and ASXL1. The multivariate Cox regression analysis showed that age and ARID1A, PIK3CA, and TP53 mutations were independent predictors of ICI therapy in HNSC patients. The main portion of the forest plot presents the hazard ratio (HR) and 95% confidence interval (95% CI), where red dots indicate *p* < 0.05. The HR indicates the predictors of favorable (HR < 1) or poor (HR > 1) OS. **(B)** Bubble plots were used to visualize the results of the univariate (left panel) and multivariate (right panel) Cox regression analyses. The blue and red dashed lines indicate *p*-values of 0.1 and 0.05, respectively. The size of the circle indicates the mutation count. The gray circles represent variables with *p* < 0.1 (left panel) or *p* < 0.05 (right panel), and the yellow circles represent the clinical characteristics of patients. The color of other circles represents the gene mutations associated with a good (orange; HR < 1) or poor (green; HR < 1) prognosis.

Somatic mutation and clinical data of the MSKCC cohort were subjected to univariate Cox regression analysis (Figure 1A, left panel), and variables with *p* < 0.1 were included in multivariate Cox regression analysis together with clinical data. The results of the multivariate Cox regression analysis (Figure 1A, right panel) found that age [HR = 0.50 (95% CI 0.28–0.87), *p* = 0.014] and mutations in ARID1A [HR = 0.13 (95% CI 0.02–0.98), *p* = 0.048], PIK3CA [HR = 0.45 (95% CI 0.23–0.89), *p* = 0.021] and TP53 [HR = 1.82 (95% CI 1.04–3.17), *p* = 0.035] were independent predictors of the efficacy of ICIs. As shown in Figures 1A,B, we speculated that age > 65 years and mutations in PIK3CA and ARID1A were favorable predictors of ICI treatment (HR < 1), and the TP53 mutation was an adverse predictor (HR > 1).

**FIGURE 2:**
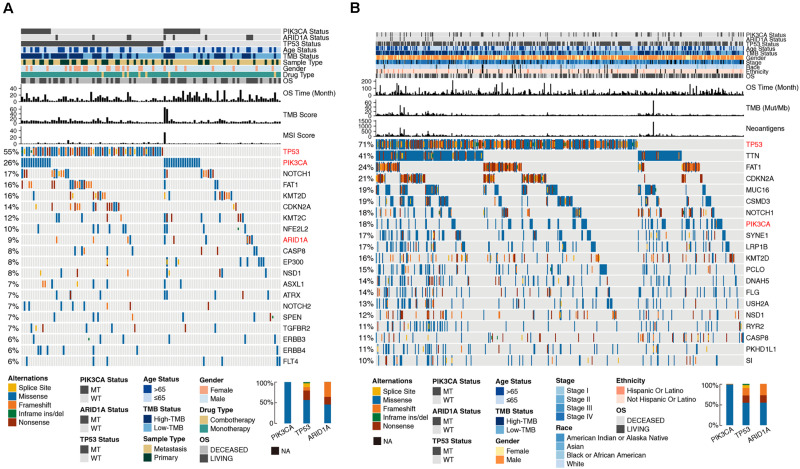
Genomic profiles of HNSC patients in the MSKCC **(A)** and TCGA **(B)** cohorts. The top 20 genes with the highest mutation frequencies and the corresponding clinical information are shown in the figure. The top five genes with the highest mutation frequencies in the MSKCC cohort were TP53 (55%), PIK3CA (26%), NOTCH1 (17%), FAT1 (16%), and KMT2D (16%). The top six genes with the highest mutation frequencies in the TCGA cohort were TP53 (71%), TTN (41%), FAT1 (24%), CDKN2A (21%), MUC16 (19%), and CSMD3 (19%). Among the three genes selected from the multivariate Cox regression analysis, TP53 had the highest mutation rate in both clinical cohorts (55 and 71%, respectively), followed by PIK3CA (26 and 18%, respectively), and ARID1A (9 and 4%, respectively). The alteration types of TP53 were dominated by missense, nonsense and frameshift mutations. Missense mutations were the main mutation type of PIK3CA, and nonsense and frameshift mutations were the main mutation types of ARID1A. The mutation types are indicated as follows: yellow indicates splice sites, blue indicates missense mutations, orange indicates frameshift mutations, green indicates inframe insertion/deletion, and brown indicates nonsense mutations. The mutation status of ARID1A, PIK3CA, and TP53, TMB status, MSI score, neoantigen status, OS, and other clinical characteristics are shown as patient annotations (upper barplot). The left barplot marks the mutation rate of each gene. Genes marked in red represent the three genes screened from the multivariate Cox regression analysis. In the figure legends, “MT” represents patients with a certain gene mutation, and “WT” represents patients without certain gene mutation.

Previous studies provided ample evidence that TMB was a predictor of sensitivity to ICI treatment (Chan et al., 2019). A high TMB is related to a high level of tumor neoantigens that can be recognized by T cells, which leads to strong antitumor immunological effects after blockade of immune checkpoints. Figure 1B shows that patients with PIK3CA, ARID1A, or TP53 mutations had high mutation counts. Although the mutation count of patients older than 65 years was relatively low, it was close to patients with a mutation count > 7. In accordance with these results, the gene mutation landscape (Figure 2) showed that PIK3CA mutant-type (MT) patients were associated with a higher TMB and better OS compared to wild-type (WT) patients, and TP53-MT patients were associated with a higher TMB. Notably, the OS of TP53-MT patients were significantly shorter than TP53-WT patients, which was investigated in the subsequent analysis.

The gene mutation landscape (Figure 2) showed the alteration types and clinical information of the top 20 genes with the highest mutation frequencies in the two clinical cohorts. The mutation sites of ARID1A, PIK3CA, and TP53 are shown in Supplementary Figure S2.

### KM Survival Analysis

**FIGURE 3:**
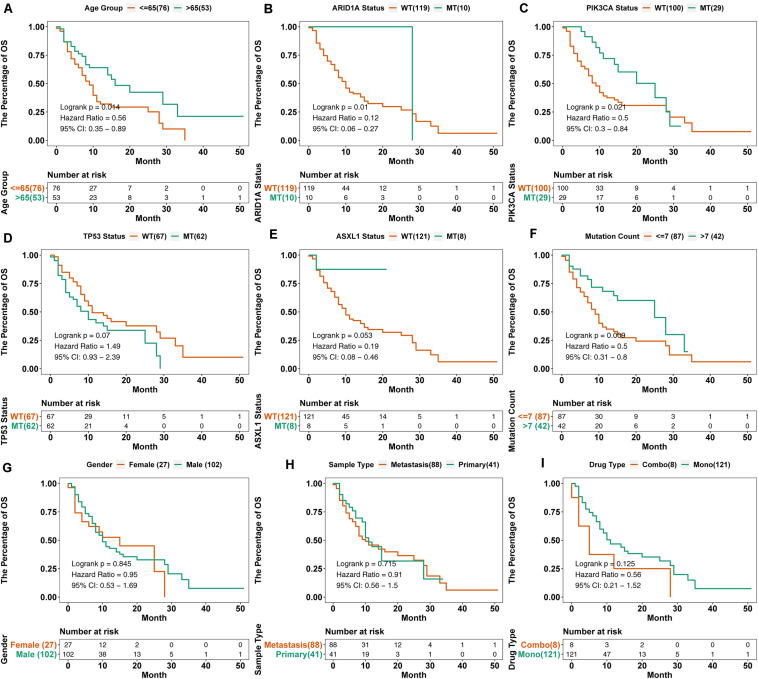
KM survival curves for OS in 129 HNSC patients from the MSKCC cohort. We performed KM survival analysis on different subgroups of patients based on age **(A)**, gene mutation status **(B–E)**, mutation count **(F)**, sex **(G)**, sample type **(H)**, and drug type **(I)**. “Combo” represents the combination treatment of anti-PD-(L)1 and anti-CTLA-4 therapy, and “Mono” represents anti-PD-(L)-1 monotherapy.

To verify the predictive value of age and mutations in PIK3CA, ARID1A, and TP53, we performed KM survival analysis on nine variables (Figure 3). Consistent with the results of the multivariate regression analysis, the OS of HNSC patients with ARID1A-MT [log-rank test, HR = 0.12 (95% CI 0.06–0.27), *p* = 0.01, Figure 3B] or PIK3CA-MT [log-rank test, HR = 0.5 (95% CI 0.3–0.84), *p* = 0.021, Figure 3C] was longer than patients with ARID1A-WT or PIK3CA-WT, respectively. Although no significant differences were observed between patients with TP53-MT and TP53-WT [log-rank test, HR = 1.49 (95% CI 0.93–2.39), *p* = 0.070, Figure 3D] or ASXL1-MT and ASXL1-WT [log-rank test, HR = 0.19 (95% CI 0.08–0.49), *p* = 0.053, Figure 3E], there was a trend toward longer OS in patients with TP53-WT. Consistent with these findings, PIK3CA and ARID1A mutations were associated with favorable outcomes, as shown in the alluvial diagram (Supplementary Figure S3).

In addition to the mutations in related genes, we also included the clinical data of patients in the survival analysis. The results showed that patients over 65 years had prolonged OS [log-rank test, HR = 0.56 (95% CI 0.35–0.89), *p* = 0.014, Figure 3A], and patients with a mutation count > 7 also benefited from ICI treatment [log-rank test, HR = 0.5 (95% CI 0.31–0.8), *p* = 0.009, Figure 3F]. Other clinical factors, such as sex, drug type, and sample type, had no statistical significance on OS.

### Immune-Related Analysis

To investigate the mechanism underlying the predictive value of age and related gene mutations, we performed tumor immunogenicity analysis on the data from the TCGA cohort and compared differences in the expression of immune checkpoint molecules, TMB and neoantigen load between subgroups based on age and gene mutations.

**FIGURE 4:**
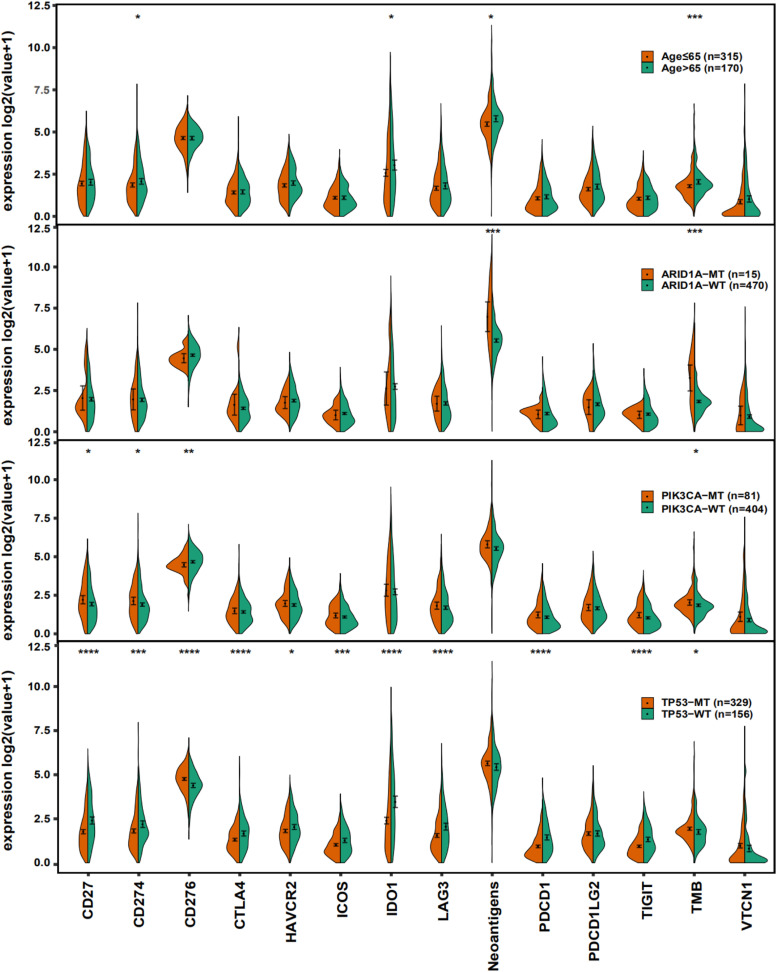
Violin plots showing the expression of immune checkpoint molecules, neoantigen load and TMB. The range of *p*-values is represented by the asterisks above each violin plot (*P ≤ 0.05; **P ≤ 0.01; ***P ≤ 0.001; ****P ≤ 0.0001).

Immune checkpoint molecules have become important molecular targets of immunotherapy because these molecules can help tumor cells escape immune system attack via immune tolerance mechanisms (Seidel et al., 2018). As shown in Figure 4, the expression levels of CD274 and IDO1, neoantigen load and TMB were higher in patients over 65 years than patients younger than 65 years (Mann Whitney U test, *p* < 0.05). Patients with ARID1A mutations had a high neoantigen load and TMB (Mann Whitney U test, *p* < 0.05). TMB and the expression of CD27 and CD274 in PIK3CA-MT patients were also higher than the PIK3CA-WT patients, while the opposite relationship was true for CD276 (Mann Whitney U test, *p* < 0.05). For TP53-MT patients, the expression levels of most of the immune checkpoint molecules listed in the figure were notably lower (Mann Whitney U test, *p* < 0.05), except CD276. We also observed a trend toward a higher TMB and neoantigen load in TP53-MT patients than TP53-WT patients (Mann Whitney U test, *p* < 0.05, *p* > 0.05, respectively), which was consistent with the results in Figure 1B.

**FIGURE 5:**
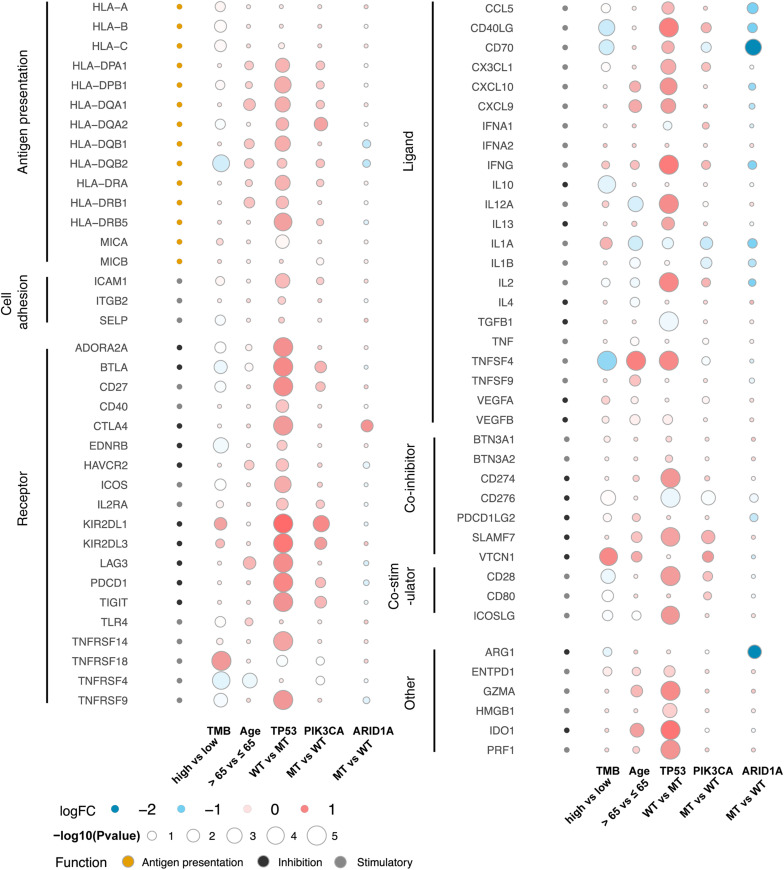
Bubble plots showing the expression of immune-related genes and immune exhaustion biomarkers. The color of the circles in the first column represents the categories of the molecules according to their functions, in which orange represents antigen presentation, black represents inhibitory molecules, and gray represents stimulatory molecules. The color of the other circles indicates logFC, as shown in the legend, and the size is proportional to the statistical significance.

To further reveal the relationships between age, gene mutations, and the tumor immune microenvironment, we assessed and compared the expression of immune-related genes and immune exhaustion biomarkers including antigen presentation-associated molecules, cell adhesion molecules, immune-related receptors and ligands, co-inhibitors, and co-stimulators between different subgroups. As shown in Figure 5, TP53 exhibited the most significant difference. Most HLA molecules were upregulated in patients with TP53-WT, PIK3CA-MT, or older than 65 years. Other immune-related molecules, such as immune-related receptors and ligands, co-inhibitors, and co-stimulators, were mostly highly expressed in TP53-WT patients. Difference in the expression of most immune-related genes between ARID1A-MT and ARID1A-WT patients was not statistically significant, but some HLA molecules, immune-related receptors, such as CCL5, CD40LG and CD70, and ARG1, were significantly downregulated in ARID1A-MT patients.

### Gene Set Enrichment Analysis (GSEA)

**FIGURE 6:**
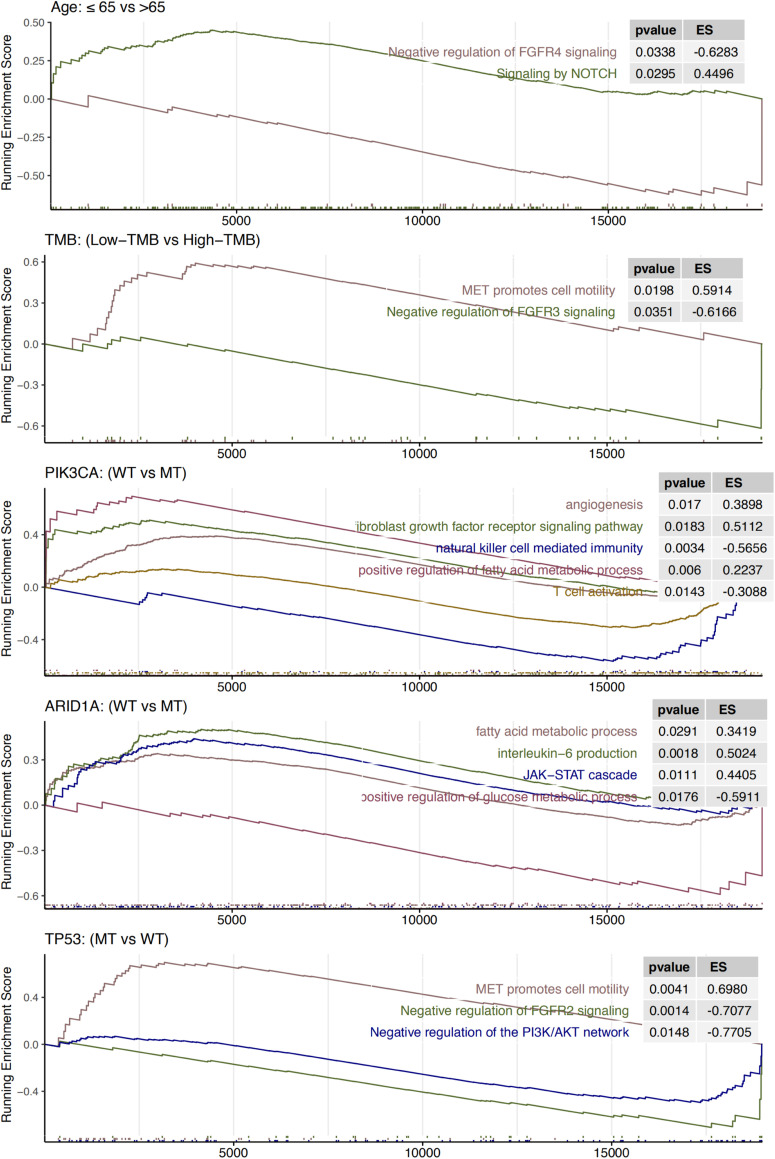
Results of gene set enrichment analysis. The following subgroups served as control groups: age > 65 years, high TMB, ARID1A-MT, PIK3CA-MT, and TP53-WT. ES > 0 indicates that the corresponding pathways are significantly enriched in the experimental groups. Colors of curves correspond to the font colors of the pathway names.

We further examined whether signaling pathways were aberrantly activated in different age and gene mutation subgroups (Figure 6). The subgroups associated with a poor prognosis (low TMB, age ≤ 65 years, PIK3CA-WT, ARID1A-WT, and TP53-MT) were enriched for FGFR, MET and other cancer-promoting pathways, such as NOTCH, JAK-STAT, PI3K-Akt, and angiogenesis. Signaling pathways involving the activation of T cells and NK cells were also enriched in PIK3CA-MT patients, and enrichment of the IL-6-related pathway was observed in ARID1A-WT patients. Notably, the pathways related to cell metabolism, such as fatty acid metabolism, were primarily enriched in the PIK3CA-WT and ARID1A-WT subgroups, and the glucose metabolism pathway was enriched in the ARID1A-MT subgroup.

## Discussion

HNSC is a group of complex malignant tumors, and the immunosuppressive tumor microenvironment plays a critical role in its occurrence and development (Oddone et al., 2009). By targeting PD-(L)1 and CTLA-4, ICIs overcome immune suppression and promote the recognition and elimination of tumor cells. Compared to traditional systemic therapy, the efficacy of ICIs is better, and the incidence of adverse reactions is lower, which provide encouraging perspectives for the treatment of HNSC. Notably, not all patients benefit from ICIs. Therefore, it is of great clinical value to identify potential biomarkers that allow the identification of HNSC patients with favorable responses and long-term benefits to ICIs. Our analyses showed that age > 65 years, TP53-WT, PIK3CA-MT, and ARID1A-MT were associated with prolonged OS in HNSC patients treated with ICIs. The activated pathways, cell metabolism, TMB, PD-L1 expression, and tumor immunogenicity led to different characteristics in the immune microenvironment, which further affected the sensitivity of HNSC patients to ICI therapy (Supplementary Figure S4).

GSEA showed that upregulation of the FGFR signaling pathway was primarily enriched in the subgroups associated with a poor prognosis, such as patients aged ≤ 65 years, patients with a low TMB, and PIK3CA-WT, or TP53-MT patients. Fibroblast growth factor (FGF) primarily regulates angiogenesis, and critical cellular behaviors, such as proliferation, survival, differentiation, and migration via binding to four transmembrane tyrosine kinase receptors (FGFR1-4) of the FGFR family (Turner and Grose, 2010). Aberrant FGF signaling is generally associated with a poor prognosis (Turner and Grose, 2010). Palakurthi et al. (2019) suggested that FGFR inhibitor or anti-PD-1 antibody monotherapy would not achieve a significant survival benefit in a lung cancer mouse model, but the combination of FGFR inhibitor and anti-PD-1 therapy would significantly improve survival in mice. The synergistic effect of FGFR inhibitors and anti-PD-1 therapy may be related to the tumor immunosuppressive microenvironment induced by FGFR activation. After inhibition of the FGFR pathway, infiltrating T lymphocytes and NK cells increase, and regulatory T Cells (Tregs), tumor-associated macrophages (TAMs), myeloid-derived suppressor cells (MDSCs), T cells with an exhausted phenotype and the expression of PD-L1 decreased (Liu et al., 2014; Palakurthi et al., 2019). Our study showed that the FGFR4 signaling pathway was primarily enriched in HNSC patients aged ≤ 65 years. Whether age maybe considered a predictor of the efficacy of ICIs remains controversial. Kugel et al. (2018) showed that melanoma patients > 60 years old had a better response to anti-PD-1 treatment than patients < 60 years old, and the mechanism may be related to the increase in Treg cells and the decrease in CD8^+^ T cells in younger patients. Treg depletion increased the response of young mice to the anti-PD-1 antibody. By combining these results with the current study, we speculated that FGFR4 pathway downregulation in HNSC patients over 65 years old mediated a good response to immunotherapy via the decreased expression of PD-1 and changes of the proportion of immune cell subsets. The NOTCH signaling pathway was also enriched in HNSC patients aged ≤ 65 years. A considerable number of studies indicated that upregulation of the NOTCH pathway promoted the proliferation and migration of tumor cells and inhibited their apoptosis (Lin et al., 2010; Zou et al., 2016). Notably, Weaver et al. (2016) found that enhanced NOTCH signaling upregulated the expression of FGF1 in oral squamous cells to promote tumor migration and invasion. In addition to the FGFR signaling pathway, the PIK3CA-WT subgroup was also enriched for the angiogenesis signaling pathways and downregulated signaling pathways involving NK cell-mediated immunity and T cell activation. This result was also consistent with the results from prior studies on the effect of FGFR activation on angiogenesis (Turner and Grose, 2010) and immune cell subsets (Liu et al., 2014; Palakurthi et al., 2019).

Cell metabolism, including glucose and fatty acid metabolism, has an important effect on the tumor immune microenvironment. Previous studies showed that the expression of PD-L1 promoted glycolysis in tumor cells (Chang et al., 2015). The competitive uptake of glucose by tumor cells and the expression of PD-1 contribute to the metabolic shift of T cells from glycolysis (Chang et al., 2015) to fatty acid metabolism (Patsoukis et al., 2015). Aerobic glycolysis is necessary for T cells to differentiate into effector cells (Cham et al., 2008), whereas M2 macrophages rely on fatty acid metabolism (Huang et al., 2014), which may protect them from the effects of glucose metabolism in tumor cells. The fatty acid metabolic pathway was primarily enriched in the ARID1A-WT and PIK3CA-WT subgroups, which were associated with a poor prognosis, and the glucose metabolism pathway was enriched in the ARID1A-MT subgroup, which indicates that the function of immune cells in the ARID1A-MT subgroup was suppressed and accompanied by the expression of PD-1. Therefore, ICI treatment may change the metabolism of tumor cells and T cells and relieve the inhibition of the antitumor effect of T cells, which allows patients with ARID1A and PIK3CA mutations to gain clinical benefits.

Numerous studies demonstrated that a high TMB and high PD-L1 expression were conducive for immune cells recognition and elimination of tumor cells, which makes patients sensitive to ICI therapy (Reck et al., 2016; Chan et al., 2019; Liu et al., 2019). Some researches revealed a better response to immunotherapy in HNSC patients with high TMB (Hanna et al., 2018) or high PD-L1 expression (Ferris et al., 2016). This observation was also confirmed in our survival analysis. For example, the subgroup associated with a good prognosis (i.e., age > 65 years and ARID1A-MT, and PIK3CA-MT) had a significantly high TMB and neoantigen load and high PD-L1 expression. PIK3CA is an important oncogene in the PI3K signaling pathway (German et al., 2013), and ARID1A is a tumor suppressor gene. Most ARID1A mutations are inactivating mutations (Wu et al., 2014). Mutations in the PI3K pathway and the loss of ARID1A expression may lead to defective DNA repair in different ways and result in an increased TMB and neoantigen load (Lui et al., 2013; Shen et al., 2018). There is a positive correlation between the loss of ARID1A expression and MSI (Li et al., 2019). The deletion of ARID1A and the activation of PIK3CA may upregulate the expression of PD-L1 via activation of the PI3K/AKT pathway (German et al., 2013; Kim et al., 2019). Therefore, the favorable response of patients with ARID1A or PIK3CA mutations may be explained from the perspectives of TMB and PD-L1 expression.

Some controversy about the predictive role of TP53 mutation in immunotherapy exists, which may result from intertumoral heterogeneity. Xiao et al. (2018) showed that TP53 mutation was associated with poor clinical outcomes in patients with metastatic melanoma treated with anti-CTLA-4 therapy. Another study observed improved OS in non-small cell lung cancer patients with TP53 mutation receiving anti-PD-1 therapy (Assoun et al., 2019). Our analysis showed that HNSC patients with TP53-MT exhibited a poor response to ICIs. Compared to TP53-WT patients, TP53-MT patients exhibited a higher TMB, but the expression of most immune checkpoint molecules, such as CD27, CD274, CTLA4, HAVCR2, ICOS, IDO1, LAG3, PDCD1, and TIGIT, was decreased. Most of the HLA molecules, immune-related receptors and ligands, co-inhibitors, and co-stimulators were significantly overexpressed in TP53-WT patients. All of these results indicate the low immunogenicity of TP53-MT patients, which may be one of the mechanisms contributing to their low sensitivity to ICIs. TP53-MT patients also exhibited enriched MET, FGFR2, and PI3K/AKT signaling pathways, which promote tumor development. In a variety of tumors, including HNSC, the abnormal activation of mesenchymal-epithelial transition (MET) tyrosine kinase receptor promotes the proliferation, invasion, migration, and epithelial-mesenchymal transformation of tumor cells (Rothenberger and Stabile, 2017; Vsiansky et al., 2018; Moosavi et al., 2019). Activation of the HGF/MET signaling pathway also inhibits the function of T cells and antigen-presenting cells and induces an increase in MDSCs and Tregs (Benkhoucha et al., 2010; Yen et al., 2013).

In summary, we screened four independent prognostic predictors of the response to ICIs by performing a bioinformatics analysis of MSKCC and TCGA data. The impact of TP53 mutation on immunotherapy outcome is controversial, and it was not investigated in HNSC. As a preliminary examination, we found that TP53 mutation was a negative prognostic predictor in our study. We also identified three other positive predictors: age > 65 years, PIK3CA-MT, and ARID1A-MT. However, there are some limitations to our study, which lie primarily in the following aspects. First, the MSK-IMPACT panel was used in the MSKCC cohort, and whole-exome sequencing was used in the TCGA cohort. The number of genes included in the MSK-IMPACT panel was less than 500, and many high-frequency genes associated with HNSC revealed by whole-exome sequencing did not appear in the MSK-IMPACT panel, which resulted in the omission of some potential predictors. Second, a subset of patients in the MSKCC cohort had received other treatments before immunotherapy, which may have affected the results of the subsequent analysis. Our results must be further validated, and a more detailed mechanism must be studied.

## Data Availability Statement

The original contributions presented in the study are included in the article/Supplementary Material, further inquiries can be directed to the corresponding author/s.

## Author Contributions

YZ participated in the interpretation of data and the writing of manuscript. AL acquired and analyzed the patient data. HM, PL, and JZ made substantial contributions to the conception and design of the work. YL and WD were involved in the manuscript editing and the supervision of the entire work. All authors read and approved the final manuscript.

## Conflict of Interest

The authors declare that the research was conducted in the absence of any commercial or financial relationships that could be construed as a potential conflict of interest.

## Footnotes

1 https://portal.gdc.cancer.gov/
