# Supplementary material for: Age and Mutations as Predictors of the Response to Immunotherapy in Head and Neck Squamous Cell Cancer
Source: Front Cell Dev Biol. 2020 Dec 9;8:608969. doi: 10.3389/fcell.2020.608969 (PMC7755718; doi:10.3389/fcell.2020.608969)
Supplement: Supplementary file 1 [file Image_1.PDF]

Supplementary Material

Supplementary Figures

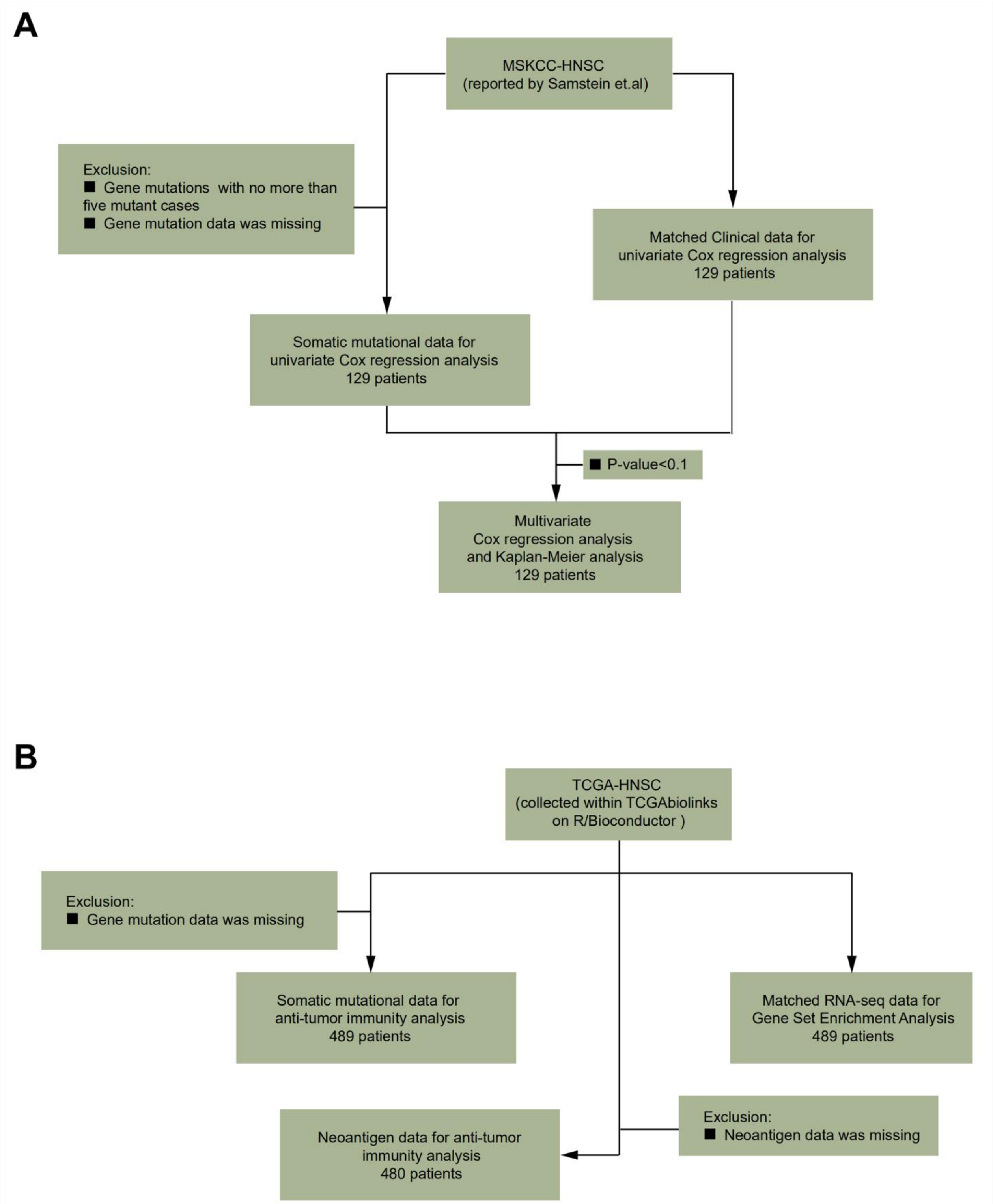

Supplementary Figure 1. Flow chart of the establishment of the clinical cohorts and subsequent analyses. (A) Establishment of the MSKCC cohort and subsequent analyses. We screened 129

HNSC patients from 138 HNSC patients who received ICI therapy reported by Samstein et al. but excluded patients without gene mutation data. Gene mutations that occurred in no more than five patients were also excluded from the subsequent analyses. (B) Establishment of the TCGA cohort and subsequent analyses. A total of 489 HNSC patients were screened after excluding patients without gene mutation data. Patients without neoantigen data were also excluded from the tumor immunogenicity analysis.

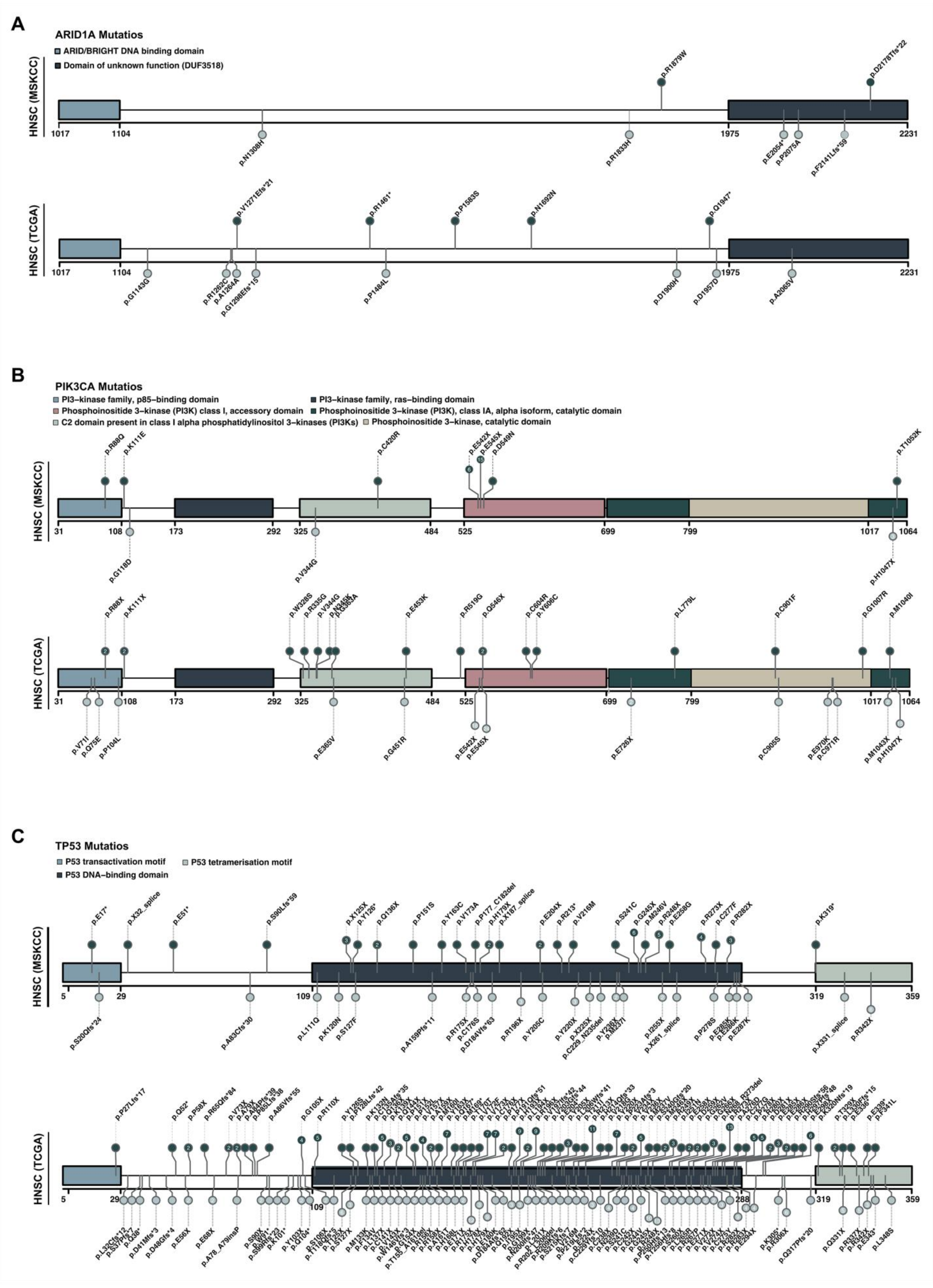

Supplementary Figure 2. Mutation sites and corresponding mutation frequencies of ARID1A (A), PIK3CA (B) and TP53 (C). The mutation sites of the TP53 gene were primarily concentrated in the p53 DNA-binding domain, of which p.R273X and p.R248X had the highest mutation frequency, followed by p.R175X, p.R213X and p.G245X. Among the mutation sites of the

PIK3CA gene, p.E545X had the highest mutation frequency, followed by p.E542X and p.H1047X. For ARID1A, the mutation sites and frequency were evenly distributed. Rectangles of different colors represent different DNA domains. The mutation sites are depicted as lollipops, and the frequency of a particular mutation is represented by the height of each lollipop, which can also be learned from the number in the corresponding circle of each lollipop.

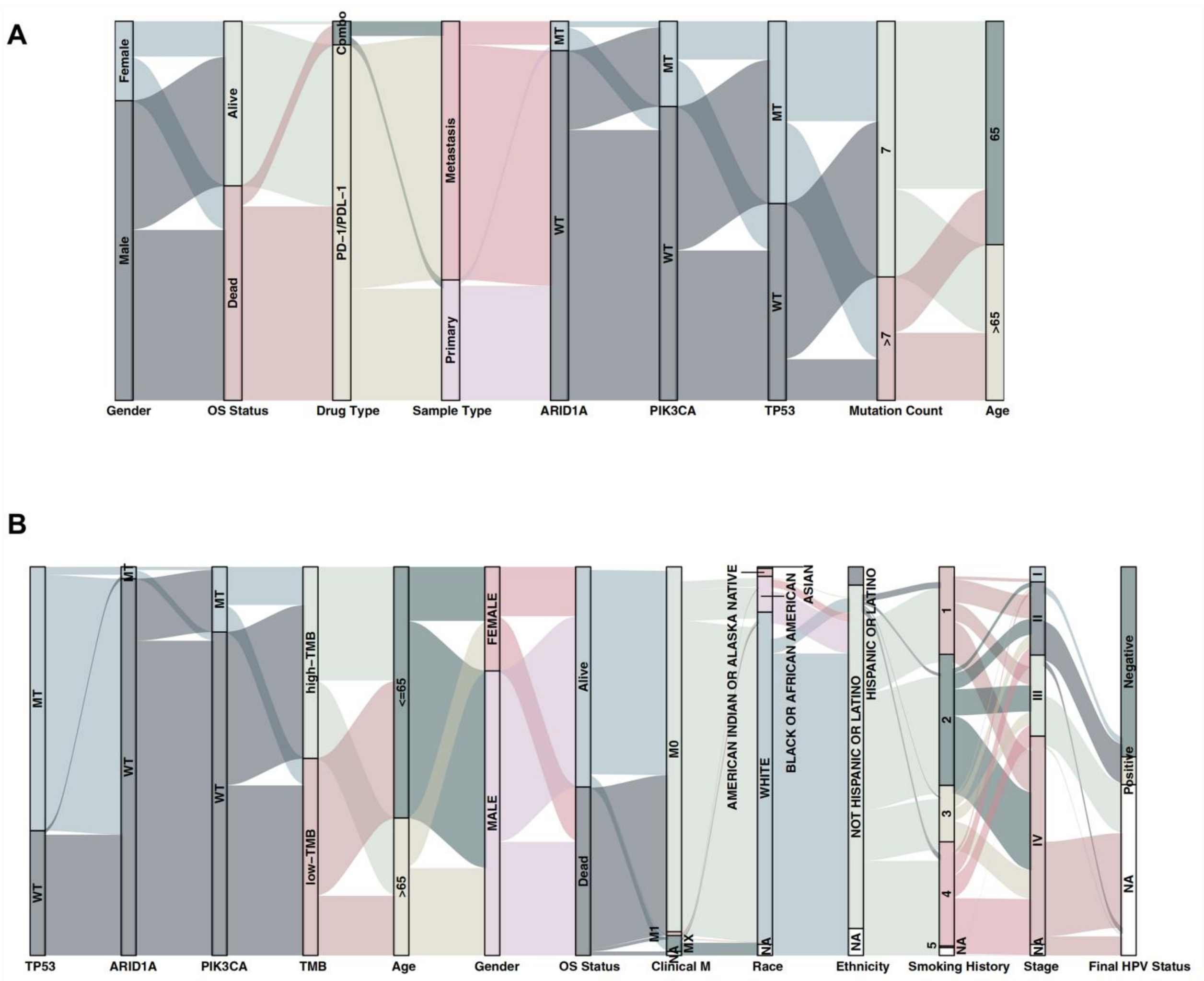

Supplementary Figure 3. Alluvial diagram of age, gene mutations, OS and other clinical characteristics. (A) Alluvial diagram derived from the data in the MSKCC cohort. (B) Alluvial diagram derived from the data in the TCGA cohort.

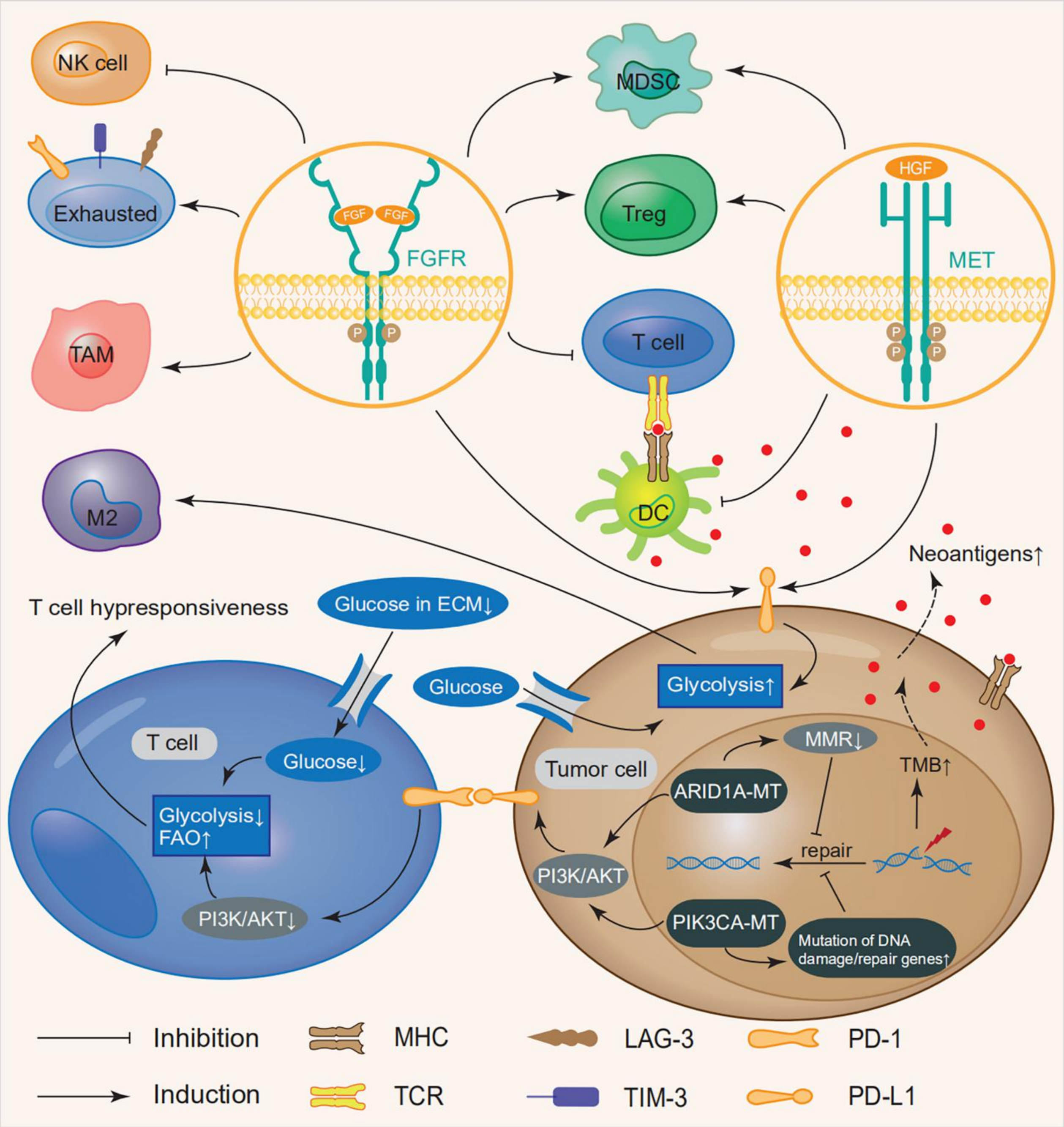

Supplementary Figure 4. The potential mechanism underlying the prognostic value of age and related gene mutations.
